# Cross-sectional quantitative analysis of the natural history of *TUBA1A* and *TUBB2B* tubulinopathies

**DOI:** 10.1038/s41436-020-01001-z

**Published:** 2020-10-21

**Authors:** Julian Schröter, Jan H. Döring, Sven F. Garbade, Georg F. Hoffmann, Stefan Kölker, Markus Ries, Steffen Syrbe

**Affiliations:** 1 Division of Pediatric Epileptology, Center for Pediatrics and Adolescent Medicine, University Hospital Heidelberg, Heidelberg, Germany; 2 Division of Neuropediatrics and Inherited Metabolic Diseases, Center for Pediatrics and Adolescent Medicine, University Hospital Heidelberg, Heidelberg, Germany

**Keywords:** *TUBA1A*, *TUBB2B*, tubulinopathy, natural history, malformations of cortical development

## Abstract

**Purpose:**

*TUBA1A* and *TUBB2B* tubulinopathies are rare neurodevelopmental disorders characterized by cortical and extracortical malformations and heterogenic phenotypes. There is a need for quantitative clinical endpoints that will be beneficial for future diagnostic and therapeutic trials.

**Methods:**

Quantitative natural history modeling of individuals with *TUBA1A* and *TUBB2B* tubulinopathies from clinical reports and database entries of DECIPHER and ClinVar. Main outcome measures were age at disease onset, survival, and diagnostic delay. Phenotypical, neuroradiological, and histopathological features were descriptively illustrated.

**Results:**

Mean age at disease onset was 4 (*TUBA1A*) and 6 months (*TUBB2B*), respectively. Mortality was equally estimated with 7% at 3.2 (*TUBA1A*) and 8.0 years (*TUBB2B*). Diagnostic delay was significantly higher in *TUBB2B* (12.3 years) compared with *TUBA1A* tubulinopathy (4.2 years). We delineated the isotype-dependent clinical, neuroradiological, and histopathological phenotype of affected individuals and present brain malformations associated with epilepsy and an unfavorable course of disease.

**Conclusion:**

The natural history of tubulinopathies is defined by the genotype and associated brain malformations. Defined data on estimated survival, diagnostic delay, and disease characteristics of *TUBA1A* and *TUBB2B* tubulinopathy will help to raise disease awareness and encourage future clinical trials to optimize genetic testing, family counseling, and supportive care.

## INTRODUCTION

As dynamic constituents of the cytoskeleton, microtubules (MTs) are pivotal during corticogenesis by mediating mitosis, cell locomotion, and formation of synaptic connections.^1,2^ MTs are mainly assembled by heterodimers of α- and ß-tubulin isotypes encoded on separate genes.^3^ According to their physiological function, an expanding number of mostly heterozygous de novo missense variants in tubulin genes have been associated with a heterogeneous group of disorders characterized by malformations of cortical development (MCDs), known as “tubulinopathies.”^4,5^ Since their first description in 2007, variants in eight genes encoding for α- (*TUBA1A*, MIM 602529; *TUBA8*, MIM 605742), ß- (*TUBB2A*, MIM 615101; *TUBB2B*, MIM 612850; *TUBB3*, MIM 602661; *TUBB4A*, MIM 602662; *TUBB*, MIM 191130), and γ-tubulins (*TUBG1*, MIM 191135) have been described.^6–13^ Tubulinopathies cause a broad spectrum of cortical and extracortical malformations with partially overlapping *TUBA1A-* and *TUBB2B*-associated phenotypes.^4,14^ Variants in either gene were identified in 6% of patients with lissencephaly.^15^ A certain isotype-specific phenotype emerged over the past years; whereas the majority of *TUBA1A*-associated tubulinopathies show severe MCDs, i.e., microlissencephaly, lissencephaly with predominant agyria, or diffuse polymicrogyria-like cortical dysplasia, *TUBB2B*-associated tubulinopathies are mainly characterized by focal (perisylvian) or generalized polymicrogyria-like cortical dysplasia.^4,14,16^ Another distinctive feature, congenital fibrosis of the extraocular muscles (CFEOM), an ocular motility disorder that has been associated with *TUBB3*, was observed in one family harboring a *TUBB2B* variant.^17,18^ In both *TUBA1A*- and *TUBB2B*-associated tubulinopathies, abnormalities of cerebellar hemispheres and vermis, basal ganglia, and commissural structures are commonly observed.^4,16,19^ Affected patients most frequently show a range of neurodevelopmental disorders including cognitive and motor impairment, abnormal muscular tone, and epilepsy resulting in a high burden of disease.^4,5,20^ Orphanet estimates a prevalence of <1:1,000,000 (tubulinopathy-associated dysgyria; Orpha: 467166).^21^ Previous studies focused on clinical, neuroradiological, and genetic features of tubulinopathies. So far, quantitative natural history data on survival rates, disease onset, and diagnostic delay are scarce but crucial for counseling of afflicted families and for design of future clinical trials with targeted therapeutic approaches. Raising disease awareness by quantitative natural history data has the potential to shorten diagnostic delay and ensure optimal supportive patient care.^22^

In this study, we therefore focused on quantification of relevant clinical endpoints such as survival, disease onset, and diagnostic delay. In addition, we describe cardinal features at disease onset and characterize associated epilepsies to improve the understanding of the clinical course and to complement therapeutic concepts for affected individuals.

## MATERIALS AND METHODS

This study was conducted in compliance with STrengthening the Reporting of OBservational studies in Epidemiology (STROBE) criteria.^23^

### Literature review and definition of variables

We performed a comprehensive literature research on PubMed using the keywords “TUBA1A,” “TUBB2B,” and “tubulinopathy.” Selected reports were published between January 2007 and July 2019. Reports were checked for duplicate mentions and verified by listed variants in the Human Gene Mutation Database at the University of Cardiff (http://www.hgmd.cf.ac.uk/ac/gene.php?gene=TUBA1A/TUBB2B). We additionally included entries from the databases ClinVar^24^ and DECIPHER^25^ providing clinical information on *TUBA1A* and *TUBB2B* missense variants depicted as “pathogenic” or “likely pathogenic.” Variants without corresponding clinical information were excluded from further analysis. According to the Human Genome Variation Society (HGVS) recommendations, *TUBA1A* and *TUBB2B* variants were standardized to the NM_006009.3 and NM_178012.4 transcript of the GRCh37/hg19 human reference genome, respectively, using the Mutalyzer Nomenclature Checker web tool.^26^ Cut-off date for data analysis was 1 July 2019. The following variables were extracted: age and symptoms at disease onset, cardinal symptoms leading to diagnosis, age at diagnosis, last reported age, information on whether the patient is alive or deceased, gestational age at birth or termination of pregnancy (TOP), age at seizure onset, main seizure types, applied antiepileptic drugs (AEDs), neuroradiological and histopathological findings, year of publication, and origin of the patient. If not explicitly stated in the report, the country of the patient’s origin has been attributed to the country of the first author’s institutional affiliation in the corresponding case description. If temporal information was expressed in semiquantitive terms, we used the following definitions: “at birth” = day 1, “newborn period” = 1 month, “infancy” = 1 year. If the individual’s decease was not explicitly stated, individuals were considered alive at the last reported age. In statistical analysis, the term “dead” is used for individuals who died due to TOP in utero or disease complications during lifetime, and was stratified as “severe course of disease” in association analysis. In detail, the main category “symptoms at disease onset” comprised eight major clinical signs. The main category “clinical features” was grouped in subcategories as follows: “epilepsy,” “microcephaly,” “developmental delay,” “motor impairment,” “abnormal muscular tone,” “further neurological features,” “musculoskeletal features,” “ocular features” including ocular motility disorders, and “facial dysmorphisms.” Cortical and extracortical findings derived from neuroradiological analyses via brain ultrasound and/or magnetic resonance image (MRI) as well as neuropathological analyses delineated in clinical reports and database entries were extracted, allocated to the affected brain region, and categorized by known tubulinopathy-associated, cortical and extracortical malformations comprising 12 and 26 features, respectively. Cohorts were subdivided whether individuals were reported as alive, deceased, or fetus after TOP. Severity stratification of the clinical variable “motor impairment” was oriented to the revised Gross Motor Function Classification System (GMFCS)^27^ as follows: none (no limitations), mild (GMFCS I), moderate (GMFCS II–III), and severe (GMFCS IV–V).

### Statistical analysis

Techniques of descriptive statistics were applied as previously reported.^28,29^ Quantitative variables are illustrated by sample size, mean, and standard deviation. Statistical comparisons between means of the two cohorts were performed with Welch’s two-sample *t*-test. A paired *t*-test was used to compare head sizes at birth and at last follow-up of the same individual. Frequencies of descriptive variables are depicted with number, sample size, and percentages. Statistical comparisons of the frequency of descriptive variables between the two cohorts were conducted using the two-tailed Fisher’s exact test. Survival was assessed and defined as the time interval between birth and age at death. Data were censored at the time of the last follow-up if the individual was still alive at last follow-up according to the extracted data of the corresponding publication. Survival estimations were calculated using the Kaplan–Meier method. The log-rank test was applied to compare potential differences between subgroups. Diagnostic delay was calculated as the time interval between age at diagnosis and age at disease onset. Prevalence of clinical characteristics was compared between both tubulinopathies. All analyses were performed using R.^30^ The world maps were plotted using the R extension ggmap.^31^ Missing data were not imputed. Sensitivity analyses were not conducted. *P* values reported were two-sided, with *P* ≤ 0.05 considered statistically significant.

### Ethics statement

This study did not make use of individual patient data. All patients had been pseudonymized prior to inclusion into our study.

## RESULTS

In 63 published clinical reports (*N*_TUBA1A_ = 49; *N*_TUBB2B_ = 14) and 61 database entries (*N*_TUBA1A_ = 52; *N*_TUBB2B_ = 9), we identified 127 *TUBA1A* and 43 *TUBB2B* variants from 190 and 55 individuals, respectively. Forty-two individuals had to be excluded from statistical analysis due to missing clinical data, resulting in an overall study cohort of 203 individuals (*N*_TUBA1A_ = 155; *N*_TUBB2B_ = 48). A flow chart delineating the exclusion process is provided in Fig. S1.

### Characteristics of the study cohorts

From 144 reported cases, 120 individuals (83.3%) with *TUBA1A* tubulinopathy were born at term (gestational week [GW] 40). Termination of pregnancy (TOP) was performed in 24 cases (16.7%) around GW 28 (range: GW 21–38) due to severe brain abnormalities on prenatal ultrasound (US) and/or magnetic resonance imaging (MRI). From 47 cases with *TUBB2B* tubulinopathy, 40 individuals (85.1%) were delivered at term and TOP was induced in 7 cases (14.9%) on average in GW 28 (range: GW 16–33). Sexual distribution was balanced in both study cohorts. Details of the study cohorts are illustrated in Table S1. The country of birth is shown in Fig. S2, suggesting a predominant distribution in Western developed countries. Values from descriptive statistics are outlined in Table S2.

### Survival estimations

**Fig. 1:**
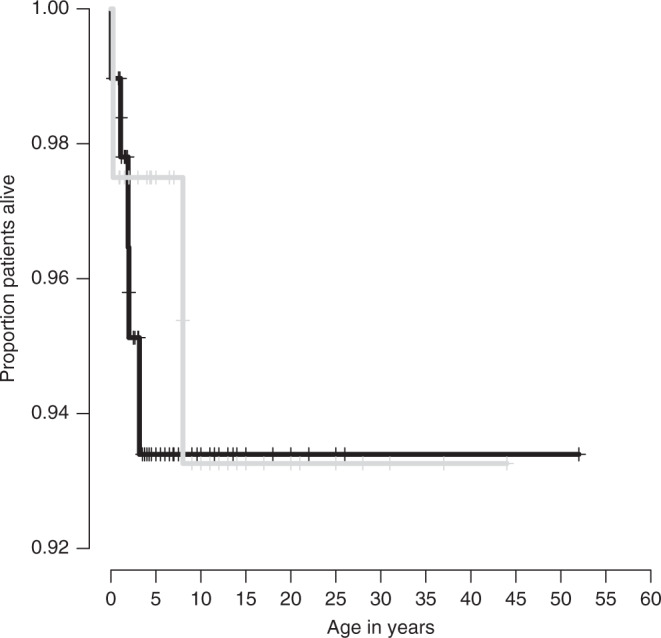
Age of onset and age at diagnosis of TUBA1A (a) and TUBB2B (b) tubulinopathy. Estimated overall survival distribution for patients with *TUBA1A* (*N* = 97, black) and *TUBB2B* tubulinopathies (*N* = 40, gray). Censored individuals are marked with a +. Log-rank test, *p* = 0.8.

In the *TUBA1A* study cohort, five individuals (3.5%) were deceased at the time of publication (Table S1). Estimated survival analysis using the Kaplan–Meier method showed that 93.4% of individuals with *TUBA1A* tubulinopathy were still alive at the age of 3.2 years (Fig. 1). In the *TUBB2B* study cohort, two individuals (4.3%) had died during follow-up and 93.3% of the individuals were alive at the age of 8.0 years (Fig. 1). Survival data showed no significant differences between both study cohorts. One individual of the *TUBA1A* cohort had died of respiratory failure at 23 months. In the *TUBB2B* cohort, one individual deceased at the age of 8 years after recurrent respiratory infections. The cause of death was not further delineated in the remaining cases.

### Quantitative and qualitative characterization of the disease onset

**Fig. 2:**
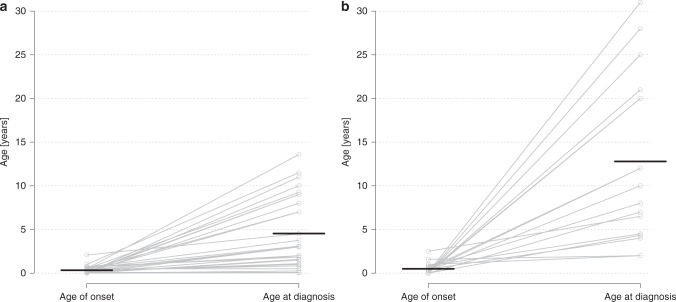
Age of onset and age at diagnosis of *TUBA1A* (**a**) and *TUBB2B* (**b**) tubulinopathy. Data were available for *N*_TUBA1A_ = 29 and *N*_TUBB2B_ = 17 individuals, respectively. Horizontal lines indicate the mean. The slopes of connecting lines represent the diagnostic delay between onset of the disease and the time of diagnosis.

We quantitatively analyzed the age at disease onset in both cohorts. In the *TUBA1A* cohort, the mean age at disease onset was 4.0 months (SD ± 5.3 months; *N* = 29), which did not differ significantly from the *TUBB2B* cohort (5.9 ± 8.2 months; *N* = 17; Table S2). In the *TUBA1A* cohort, genetic diagnosis was established at the mean age of 4.5 years (SD ± 4.0 years; *N* = 29) whereas the age at diagnosis was significantly higher in the *TUBB2B* cohort (12.8 ± 9.5 years; *N* = 17; *P* < 0.001; Table S2). Correspondingly, mean diagnostic delay after appearance of initial symptoms differed significantly between the *TUBA1A* (4.2 ± 4.0 years) and the *TUBB2B* cohort (12.3 ± 9.9 years; *P* = 0.004; Fig. 2). Early postnatal symptoms in individuals with *TUBA1A* variants were most frequently microcephaly (54.3%), seizures (42.9%), and muscular hypotonia (37.1%; Table 1). Most common, partially overlapping symptoms at disease onset in the *TUBB2B* cohort were developmental delay (47.4%), seizures (36.8%), and muscular hypotonia (21.1%). Microcephaly (54.3% vs. 15.8%) and facial dysmorphisms (31.4% vs. 0.0%) were significantly more prevalent in individuals with *TUBA1A* tubulinopathy (*P* = 0.009 and *P* = 0.005, respectively). Further symptoms at disease onset are shown in Table 1.

**Table 1:** Symptoms at disease onset in the study cohorts.

Symptoms at disease onset	*TUBA1A*	*TUBB2B*	*P* value
Microcephaly	19/35 54.3%	3/19 15.8%	0.009^a^
Facial dysmorphism	11/35 31.4%	0/19 0.0%	0.005^a^
Developmental delay	10/35 28.6%	9/19 47.4%	0.234
Muscular hypotonia	13/35 37.1%	4/19 21.1%	0.358
Seizures	15/35 42.9%	7/19 36.8%	0.775
Feeding difficulties	5/35 14.3%	0/19 0.0%	0.149
Poor visual contact	3/35 8.6%	1/19 5.3%	1.0
Nystagmus	2/35 5.7%	0/19 0.0%	0.535
Other	4/35 11.4%	4/19 21.1%	0.431

Proportions are illustrated in numbers (top) and percentages (bottom).

^a^Significant difference.

### Clinical features in the course of disease

*TUBA1A*- and *TUBB2B*-associated disease features are comprehensively outlined in Table 2 and Table S3. During the course of disease, motor impairment, developmental delay, abnormalities of the muscular tone, microcephaly, and epilepsy were the most frequent clinical features but differed in distribution between individuals with *TUBA1A* and *TUBB2B* tubulinopathy. Gross motor function was more commonly affected in the *TUBA1A* (97.3%) than in the *TUBB2B* cohort (73.1%; *P* = 0.001) whereas normal motor function was significantly more prevalent in the *TUBB2B* cohort (19.2% vs. 2.9%; *P* = 0.012). Mild, moderate, and severe motor impairment was equally distributed in both cohorts. All deceased patients in the *TUBA1A* cohort had severe motor impairment. Accordingly, abnormalities of the muscular tone such as spastic di- or tetraplegia, muscular hypo- and dystonia occurred more frequently in the *TUBA1A* cohort (89.6% vs. 48.0%; *P* < 0.001). Occurrence of musculoskeletal comorbidities was likewise distributed in both cohorts (11.9% vs. 35.3%; *P* = 0.061). The vast majority of individuals showed delay in cognitive, speech, and motor development. In most cases, more than one developmental area was affected. In the *TUBA1A* cohort, delay of global development (95.7% vs. 76.7%; *P* = 0.005), speech (98.9% vs. 88.6%; *P* = 0.020), and motor development (97.8% vs. 83.8%; *P* = 0.007) were significantly more common than in the *TUBB2B* cohort where mild cognitive disability such as isolated learning difficulties were more prominent (1.1% vs. 14.3%; *P* = 0.006). Microcephaly had a similar prevalence in both cohorts (74.3% vs. 67.4%). In contrast, primary microcephaly occurred more frequently in the *TUBA1A* (88.2%) than in the *TUBB2B* cohort (50.0%; *P* = 0.012) and reduction of the occipitofrontal diameter (OFD) was pronounced in both groups at last follow-up (−3.7 and −3.5 SD, respectively). Microcephaly in *TUBA1A* tubulinopathy was progressive from −2.0 SD at birth compared with −4.1 SD at last follow-up in cases with both reported variables (*N* = 9; *P* = 0.004). In the *TUBB2B* cohort, data on OFD at birth was only available in one case. Epilepsy was common in both cohorts: 65.9% (*TUBA1A*) and 54.8% (*TUBB2B*) of individuals developed seizures during the observation period. The mean age of seizure onset was 7.5 months (*N*_*TUBA1A*_ = 25) and 33.1 months (*N*_TUBB2B_ = 14; *P* = 0.331; Table S2). In both cohorts, infantile onset of epilepsy was seen in the majority of cases (84.6% vs. 78.6%) indicating a broader range of age at seizure onset in *TUBB2B* cases. Infantile spasms were the most frequent seizure type in both cohorts (32.5% vs. 27.3%). Individuals generally showed a broad range of focal and generalized semiologies that occurred similarly in *TUBA1A*- and *TUBB2B*-associated epilepsy. Epilepsy was more often refractory to treatment in the *TUBA1A* (64.7%) than in the *TUBB2B* cohort (37.5%, *P* = 0.235). Valproic acid, phenobarbital, levetiracetam, carbamazepine, and topiramate were the most frequently used antiepileptic drugs (AEDs). Further clinical features were facial diplegia (21.7%), which was exclusively observed in the *TUBA1A* cohort, and ocular abnormalities, which were described in 58.6% (*TUBA1A*) and 65.2% (*TUBB2B*) of the individuals, respectively. Strabismus and nystagmus were the most common ocular motility disorders. Congenital fibrosis of the extraocular muscles (CFEOM) was exclusively depicted in the *TUBB2B* cohort. Facial dysmorphisms were generally found in both cohorts but were more commonly reported in *TUBA1A* (68.8%) than in *TUBB2B* fetuses (20.0%) and primarily included retrognathism (19.0%) and hypertelorism (11.4%), which were not present in the *TUBB2B* cohort where individuals primarily showed downturned corners of the mouth (0.0% vs. 33.3%). Reported facial dysmorphisms are delineated in Table S4.

**Table 2:** Classification and outcome of epilepsy in the study cohorts.

	*TUBA1A*	*TUBB2B*	*P* Value
Classification of epilepsy			
Focal	13/40 32.5%	3/11 27.3%	1.0
Generalized	12/40 30.0%	6/11 54.5%	0.165
Infantile spasms	13/40 32.5%	3/11 27.3%	1.0
Specific semiologies			
Absence	2/40 5.0%	0/11 0.0%	1.0
Tonic	4/40 10.0%	2/11 18.2%	0.598
Clonic	2/40 5.0%	0/11 0.0%	1.0
Tonic–clonic	8/40 20.0%	2/11 18.2%	1.0
Myoclonic	5/40 12.5%	0/11 0.0%	0.572
Other	4/40 10.0%	2/11 18.2%	0.598
Epilepsy outcome	54/82 65.9%	17/31 54.8%	0.286
Intractable/refractory seizures	22/34 64.7%	3/8 37.5%	0.235
Occasional/controlled seizures	12/34 35.3%	5/8 62.5%	0.235
Not assessed	20/54 37.0%	9/17 52.9%	–

Prevalence and course of epilepsy, seizures types, and outcome are listed. Proportions are illustrated in numbers (top) and percentages (bottom).

### Neuroanatomical and histopathological features

**Fig. 3:**
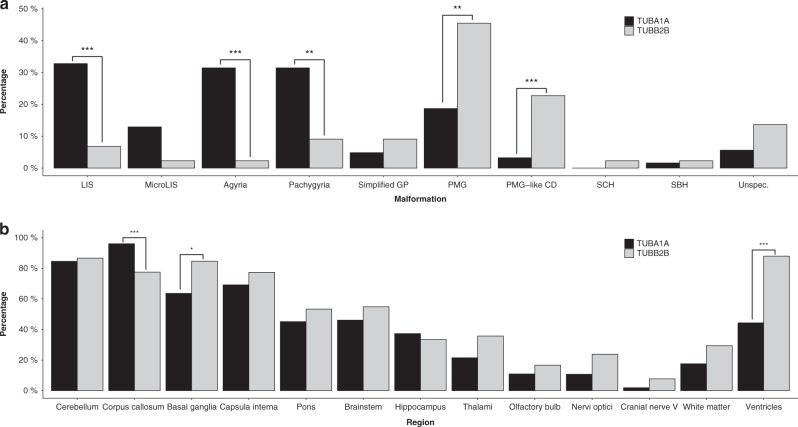
Frequency of cortical (**a**) and extracortical findings (**b**) in the *TUBA1A* (black) and *TUBB2B* (gray) cohorts. Statistically significant differences are marked with asterisks. *LIS* lissencephaly, *MicroLIS* microlissencephaly, *PMG* polymicrogyria, *PMG-like CD* polymicrogyria-like cortical dysplasia, *SBH* subcortical band heterotopia, *SCH* schizencephaly, *Simplifed GP* simplified gyral pattern, *Unspec.* unspecified. Level of significance: * ≤ 0.05, ** ≤ 0.01, *** ≤ 0.001.

A detailed description of cortical and extracortical malformations sorted by subgroups is presented in Fig. 3, Tables S5, S6, and Figs. S3, S4.

Cortical malformations were reported in 89.7% and 97.8% of individuals in the *TUBA1A* and *TUBB2B* cohort. They were present in all deceased individuals and fetuses. Lissencephaly (32.8% vs. 6.8%) with pachygyria and/or agyria was a distinctive cortical finding in *TUBA1A* compared with *TUBB2B* tubulinopathy (*P* < 0.001; Fig. 3; Table S5). Microlissencephaly as the most severe form of the lissencephaly spectrum was more prevalent in the *TUBA1A* cohort and was reported in 50.0% of fetal cases whereas only one fetal case was described in the *TUBB2B* cohort. Simplified gyral pattern as milder form of the spectrum was exclusively described in living individuals and evenly distributed (Fig. 3; Table S5). Polymicrogyria (PMG) and polymicrogyria-like cortical dysplasia (PMG-like CD) were significantly more frequent in individuals of the *TUBB2B* cohort (*P* = 0.001 and *P* < 0.001, respectively; Fig. 3; Table S5). Extracortical findings were described in all but one case in the *TUBB2B* cohort. Complete cerebellar agenesis, basal ganglia hypertrophy, and brainstem dysplasia were extracortical characteristics exclusively described in the *TUBA1A* cohort. Additionally, abnormalities of corpus callosum (CC) were significantly more common in *TUBA1A* tubulinopathy (96.1% vs. 77.5%; *P* < 0.001). In return, *TUBB2B* tubulinopathy showed significant differences concerning the occurrence of basal ganglia (63.6% vs. 84.6%; *P* = 0.02) and ventricular dysgenesis, especially ventriculomegaly (44.3% vs. 88.0%; *P* < 0.001).

Consistent with the distribution pattern, statistical frequency analysis identified neuroradiological features that were significantly associated with a mild or severe course of disease and epilepsy. For example, microlissencephaly, agyria, cerebellar hypoplasia, complete agenesis of the CC, basal ganglia, and olfactory bulb, hippocampal dysplasia, and pons hypoplasia were more common in individuals of the *TUBA1A* cohort defined as severely affected (i.e., deceased or fetal cases) whereas pachygyria was more frequently found in living individuals. In the *TUBB2B* cohort, agenesis of the olfactory bulb was significantly more prevalent in severely affected individuals while multifocal PMG, PMG-like CD, and ventriculomegaly were associated with a rather milder course of disease (Fig. S3). In the *TUBA1A* cohort, epilepsy was associated with lissencephaly and agyria. In the *TUBB2B* cohort, epilepsy was associated with complete agenesis of the CC and brainstem hypoplasia (Fig. S4). Incidence of epilepsy and intractable seizures did not significantly differ in deceased individuals.

Histopathological data from clinical reports of fetal cases are summarized in Table S7. Most common findings in fetuses of the *TUBA1A* cohort were heterotopic neurons (86.4%), hypoplastic olivary nuclei (65.0%), and a disorganized corticospinal tractum (60.0%). In the *TUBB2B* cohort, heterotopic neurons (83.3%), enlarged germinal zones (66.7%), and neuroglial overmigration (50.0%) were described, with neuroglial overmigration being a distinctive histopathological feature in *TUBB2B* tubulinopathy (9.5% vs. 50.0%; *P* = 0.024).

## DISCUSSION

We quantitatively and comparatively delineate the natural history of *TUBA1A* and *TUBB2B* tubulinopathies by assessment of a large meta-cohort of 203 patients and provide their distinctive clinical features. In particular, we define hard clinical endpoints and seminal features at disease onset and during the course of disease as well as distinctive characteristics: *TUBA1A* tubulinopathy is initially characterized by primary, progressive microcephaly and facial dysmorphisms including retrognathism and hypertelorism, developing facial diplegia and abnormalities of the muscular tone in the course of disease resulting in severe motor impairment. Neuroradiological features primarily include lissencephaly and abnormalities of the corpus callosum. In comparison, patients with *TUBB2B* tubulinopathy show developmental delay at disease onset and are characterized by isolated learning difficulties and congenital fibrosis of the extraocular muscles. PMG and PMG-like CD, with their microscopic correlate of neuronal overmigration, and abnormalities of the basal ganglia and ventricles are predominant neuroradiological and histopathological features.

Both tubulinopathies showed a relevant estimated mortality of 7% during the first decade of life. Documented patients in the *TUBA1A* cohort died earlier, mainly within this vulnerable period. Even though documentation in the literature is sparse, our present data indicate respiratory complications as a main cause of death, likely being the result of severe motor impairment that was present in all deceased patients. This is in line with data from population-based studies that associated neurological impairment to premature death in children.^32^ Mortality of *TUBA1A* and *TUBB2B* tubulinopathy might be even higher as fetuses from terminated pregnancies represent a relevant percentage of reported *TUBA1A* (16.7%) and *TUBB2B* (14.9%) cases.

*TUBA1A* and *TUBB2B* tubulinopathies usually become symptomatic in infancy at an average age of 4 and 6 months, respectively, but a considerable diagnostic delay of 4.2 and 12.3 years is observed until a genetic diagnosis is established. Especially individuals with *TUBB2B* tubulinopathy were diagnosed late in childhood. Novel genetic screening tests together with experienced clinicians will likely help to establish an earlier diagnosis for these ultraorphan conditions with distinct central nervous system (CNS) features but unspecific clinical signs.^15,33,34^ In both tubulinopathies presented here, seizures and muscular hypotonia were cardinal symptoms at disease onset. Distinctive features included primary, progressive microcephaly and facial dysmorphisms in individuals with *TUBA1A* tubulinopathy. Our clinical data suggest a generally milder course of *TUBB2B* compared with *TUBA1A* tubulinopathy regarding the time course of estimated survival and the less frequent occurrence of neurological comorbidities such as motor impairment (97.3% vs. 73.1%), abnormalities of the muscular tone (89.6% vs. 48.0%), and global developmental delay (95.7% vs. 76.7%). Additionally, nearly 20% of individuals with *TUBB2B* tubulinopathy seem to have normal motor function, which is in line with previous reports of a low prevalence of muscular tone abnormalities in *TUBB2B* tubulinopathy.^4^ Diagnostic delay in *TUBB2B* tubulinopathy could be partially explained by the more subtle clinical signs at disease onset. According to the literature and derived from our data, patients with *TUBA1A* compared with *TUBB2B* tubulinopathy more frequently show severe MCDs such as microlissencephaly, lissencephaly, and agyria.^4,16^ Comparatively, less severe MCDs such as PMG and PMG-like CD, which are also seen in connatal infections, appear more specific for *TUBB2B* tubulinopathy, therefore not prompting early genetic testing.^35,36^

Epilepsy is a cardinal feature at disease onset of *TUBA1A* and *TUBB2B* tubulinopathy: 65.9% and 54.8% of patients, respectively, developed seizures during the observation period. In the majority of cases, epilepsy manifests in the first year of life with infantile spasms as the most common epilepsy syndrome. However, the spectrum of semiology and severity is broad and course of epilepsy seems to be more severe in *TUBA1A* tubulinopathy where epilepsy remained refractive in two thirds of the reported cases. We could show that particular malformations of cortical (lissencephaly, agyria) and extracortical (complete agenesis of the CC, brainstem hypoplasia) brain structures were associated with epilepsy in *TUBA1A* and *TUBB2B* tubulinopathies, respectively. Accordingly, previous studies suggested basal ganglia and cerebellar dysfunction favoring epileptogenesis.^37,38^ While some authors described the course of epilepsy in tubulinopathies as rather benign, this discrepancy to our study results is likely explained by the different cohort size.^20^

### Study limitations and future directions

Quantitative natural history modeling based on clinical reports is inherently accompanied by several relevant limitations. First, ascertainment bias affects soft variables such as clinical and neuroradiological features. In case of *TUBA1A* tubulinopathy, a previous study showed clustering of descriptive terms depending on the clinical report from which they originated.^5^ Furthermore, predominance of patients’ countries of origin could be biased by large studies executed in developed countries with sufficient resources for comprehensive genetic testing. Second, lack of a standardized study protocol leads to data loss, which is why prospective studies are more suitable for assessment of descriptive variables. Accordingly, this study focuses on hard clinical endpoints such as survival, disease onset, and diagnostic delay. Third, survival estimations are based upon small sample sizes without consideration of possible confounders such as supportive care measures. As tubulinopathies are extremely rare diseases, prospective assessment of survival data would require an international multicenter study approach with a high logistic and temporal burden. In contrast, hard clinical endpoints are provided by quantitative natural history modeling methodology in a timely manner.^29^

### Conclusions

In this present study, we define the important primary endpoints survival, disease onset, and diagnostic delay analyzing the so-far largest quantitative analysis cohort of patients with *TUBA1A* and *TUBB2B* tubulinopathy. Thereby, we provide clinical and epidemiological data that are of particular interest for future diagnostic and therapeutic trials. Moreover, we sharpen the phenotypic profile of *TUBA1A* and *TUBB2B* tubulinopathy by comprehensively delineating isotype-dependent characteristics at disease onset and in the course of disease as well as neuroradiological features associated with epilepsy and an unfavorable clinical outcome. By outlining excessive diagnostic delay of tubulinopathies, we intend to raise awareness of these ultraorphan diseases as diagnostic uncertainty and a relevant proportion of TOPs can cause high psychosocial burden for afflicted families. Earlier diagnosis, e.g., using targeted panel testing, exome sequencing, or prospectively noninvasive prenatal genetic testing following detection of fetal brain abnormalities, is pivotal to counsel parents concerning accurate prognosis and recurrence risk.^39^

## Supplementary information

Supplementary_information
