# Supplementary material for: Cross-sectional quantitative analysis of the natural history of TUBA1A and TUBB2B tubulinopathies
Source: Genet Med. 2020 Oct 21;23(3):516–23. doi: 10.1038/s41436-020-01001-z (PMC7935713; doi:10.1038/s41436-020-01001-z)
Supplement: Supplementary file 1 — Supplementary_information [file 41436_2020_1001_MOESM1_ESM.pdf]

Supplementary information -

Cross-sectional quantitative analysis of the natural history of *TUBA1A* and *TUBB2B* tubulinopathies

Schröter et al., Genetics in Medicine 2020

<u>Supplementary figure and table legends:</u>

Figure 1:

Study flow chart.

Identification of clinical reports and database entries containing sufficient clinical information for further phenotypic analysis.

Figure S2:

World map with frequency of reported *TUBA1A* (A) and *TUBB2B* (B) tubulinopathy cases.

TUBA1A (N = 139) and *TUBB2B* (N = 47) patients are highlighted in greyscale according to the frequency of reported patients in the respective country.

Figure S3:

Frequency analysis of cortical and extra-cortical features sorted by occurrence in living and dead individuals.

Feature occurrence in the *TUBA1A* and *TUBB2B* cohort, respectively, is depicted by mosaic graphs. Rectangles are highlighted when statistical estimations in the log-linear model was exceeded (blue) or undercut (red). Significance was analyzed via chi square test. Abbreviations: BG_Ag = Agenesis of the basal ganglia; cACC = Complete agenesis of the corpus callosum; Hipp_Dys = Hippocampal dysplasia;

HS_Hypo = Hemispheric (cerebellar) hypoplasia; MicroLIS = Microlissencephaly; Olfbulb_Ag = Agenesis of the olfactory bulb; PG = Pachygyria; PMGlike = Polymicrogyria-like cortical dysplasia; PMG_Multi = Multifocal polymicrogyria; Pons_Hypo = Pons hypoplasia.

Figure S4:

Frequency analysis of cortical and extra-cortical features sorted by occurrence in individuals with or without epilepsy.

Feature occurrence in the *TUBA1A* and *TUBB2B* cohort, respectively, is depicted by mosaic graphs. Rectangles are highlighted when statistical estimations in the log-linear model was exceeded (blue) or undercut (red). Significance was analyzed via chi square test. Abbreviations: BS_Hypo = Brainstem hypoplasia; cACC = Complete agenesis of the corpus callosum; cLIS = Lissencephaly.

Table S1:

Characteristics of the TUBA1A and TUBB2B study cohorts.

Table S2:

Descriptive statistics of the *TUBA1A* and *TUBB2B* cohort.

Significant differences are marked with asterisks. Abbreviations: OFD = Occipitofrontal diameter; SD = Standard deviation; TOP = Termination of pregnancy.

Table S3:

Clinical features of the *TUBA1A* and *TUBB2B* cohorts.

Significant differences are marked with asterisks.

Table S4:

Detailed facial dysmorphisms in the study cohorts.

Dysmorphism are sorted by facial region.

Table S5:

Cortical findings in the study cohorts.

Values are depicted in N (top) and Percentage of the total subgroup (bottom).

Significant differences are marked with asterisks.

Table S6

Extra-cortical findings in the study cohorts.

N (top) and Percentage of the total subgroup (bottom) are depicted. Significant differences are marked with asterisks.

Table S7:

Histopathological findings in the study cohorts.

Most frequent neuropathological findings were cortical and extra-cortical malformations whose histopathological features are further subdivided here.

Supplementary figures and tables

Figure S1

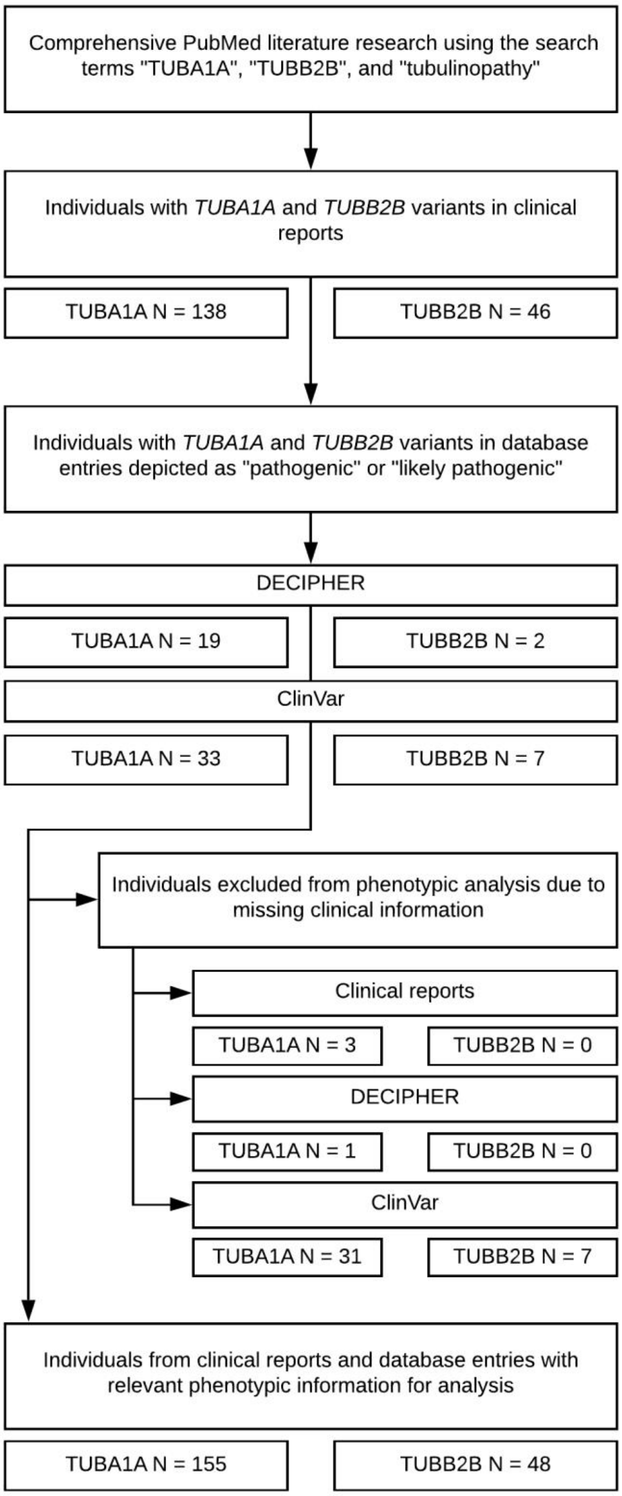

Figure S2

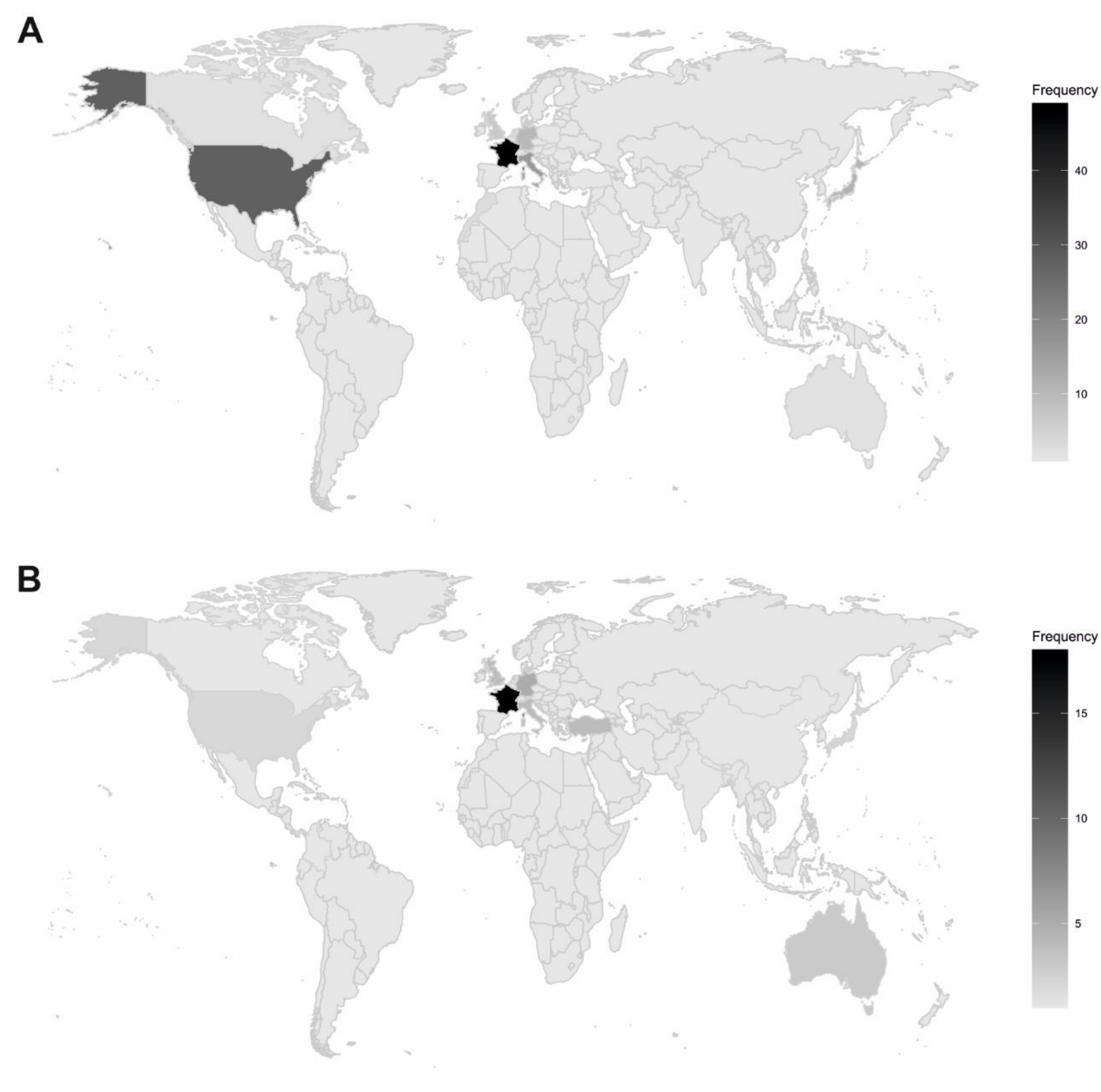

Figure S3

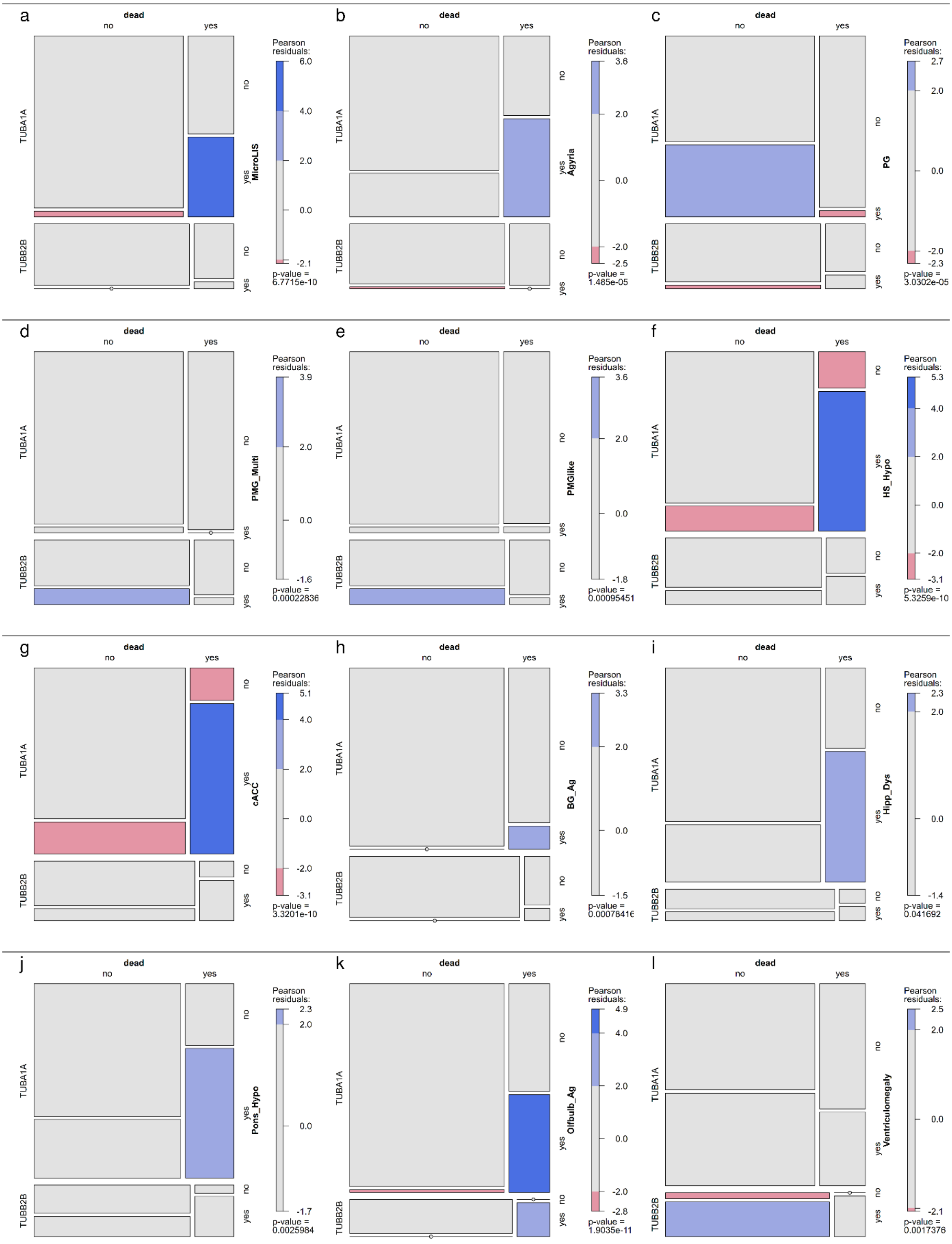

a
dead
no
yes
TUBA1A
TUBB2B
MicroLIS
Pearson residuals:
6.0
4.0
2.0
0.0
-2.1
p-value = 6.7715e-10
b
Agyria
Pearson residuals:
3.6
2.0
0.0
-2.0
-2.5
p-value = 1.485e-05
c
PG
Pearson residuals:
2.7
2.0
0.0
-2.0
-2.3
p-value = 3.0302e-05
d
PMG_Multi
Pearson residuals:
3.9
2.0
0.0
-1.6
p-value = 0.00022836
e
PMGlike
Pearson residuals:
3.6
2.0
0.0
-1.8
p-value = 0.00095451
f
HS_Hypo
Pearson residuals:
5.3
4.0
2.0
0.0
-2.0
-3.1
p-value = 5.3259e-10
g
cACC
Pearson residuals:
5.1
4.0
2.0
0.0
-2.0
-3.1
p-value = 3.3201e-10
h
BG_Ag
Pearson residuals:
3.3
2.0
0.0
-1.5
p-value = 0.00078416
i
Hipp_Dys
Pearson residuals:
2.3
2.0
0.0
-1.4
p-value = 0.041692
j
Pons_Hypo
Pearson residuals:
2.3
2.0
0.0
-1.7
p-value = 0.0025984
k
Olfbulb_Ag
Pearson residuals:
4.9
4.0
2.0
0.0
-2.0
-2.8
p-value = 1.9035e-11
l
Ventriculomegaly
Pearson residuals:
2.5
2.0
0.0
-2.1
p-value = 0.0017376

Figure S4

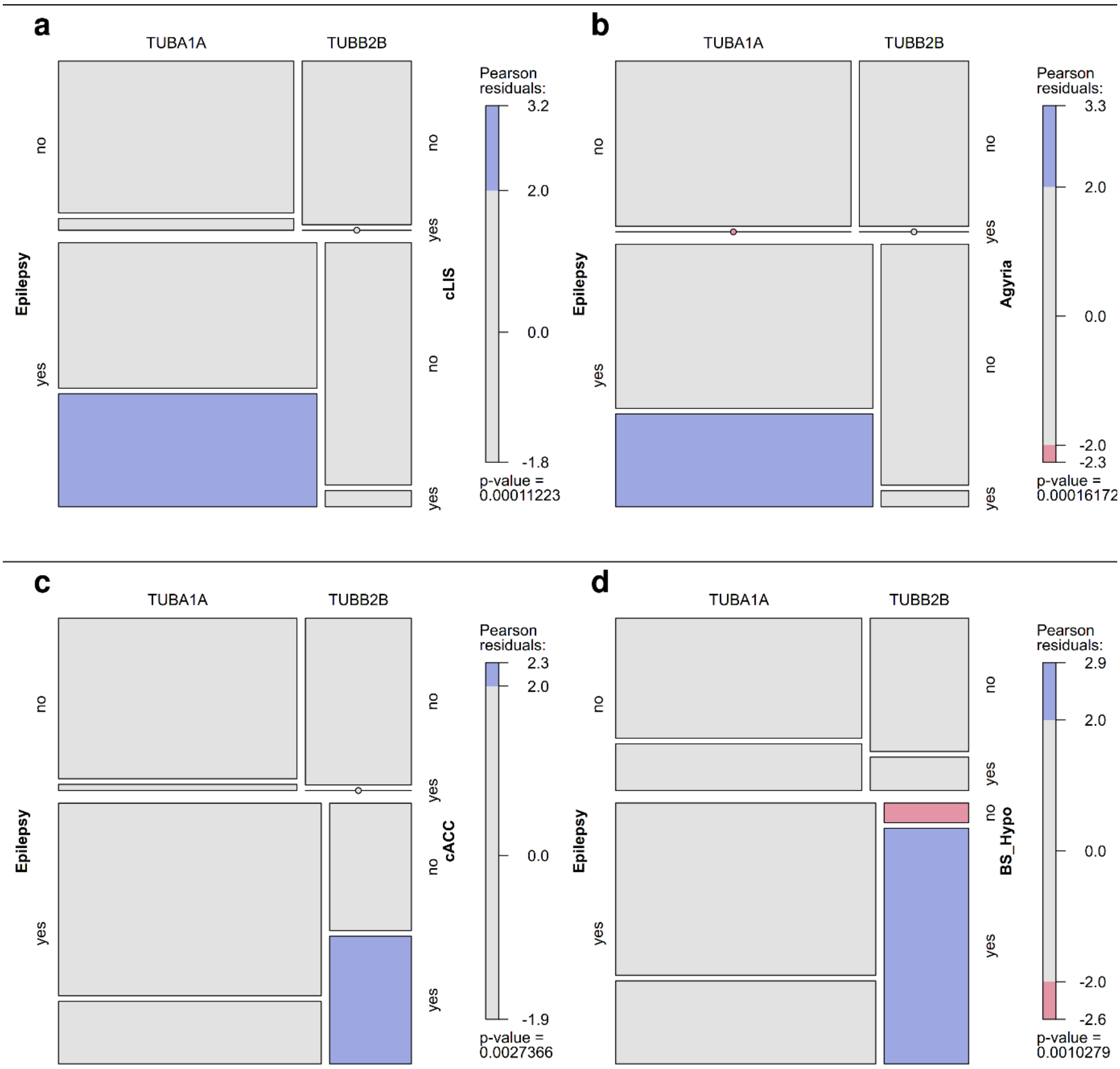
a
TUBA1A
TUBB2B
Pearson residuals:
3.2
2.0
0.0
-1.8
p-value = 0.00011223
Epilepsy
no
yes
cLIS
b
TUBA1A
TUBB2B
Pearson residuals:
3.3
2.0
0.0
-2.0
-2.3
p-value = 0.00016172
Agyria
c
TUBA1A
TUBB2B
Pearson residuals:
2.3
2.0
0.0
-1.9
p-value = 0.0027366
cACC
d
TUBA1A
TUBB2B
Pearson residuals:
2.9
2.0
0.0
-2.0
-2.6
p-value = 0.0010279
BS_Hypo

Table S1

	TUBA1A		TUBB2B	
	**N**	**Percentage**	**N**	**Percentage**
Reported variants	190	-	55	-
Reported individuals	144	-	47	-
• Alive	115/144	79.9 %	38/47	80.9 %
• Fetuses	24/144	16.7 %	7/47	14.9 %
• Deceased	5/144	3.5 %	2/47	4.3 %
• Not assessed	46/190	24.2 %	8/55	14.5 %
Sex				
• Female	46/104	44.2 %	19/40	47.5 %
• Male	58/104	55.8 %	21/40	52.5 %

Table S2

	TUBA1A		TUBB2B		
**Parameter**	**Mean ± SD**	**N**	**Mean ± SD**	**N**	**P-Value**
Age at disease onset [months]	4.0 ± 5.3	29	5.9 ± 8.2	17	0.344
Age at seizure onset [months]	7.5 ± 9.4	25	33.1 ± 94.5	14	0.331
Age at diagnosis [months]	54.4 ± 48.1*	29	153.4 ± 113.8*	17	< 0.001*
Diagnostic delay [months]	50.4 ± 47.9*	29	147.5 ± 118.4*	17	0.004*
Last reported age [months]	75.9 ± 87.7*	97	139.2 ± 126.0*	40	0.005*
Gestational week	39.6 ± 1.5	38	39.6 ± 1.0	11	0.941
Gestational week - TOP	27.3 ± 4.9	23	27.0 ± 5.7	7	0.892
Head size – at birth [SD OFD]	- 2.4 ± 1.3	14	- 3.3	1	-
Head size – last follow-up [SD OFD]	- 3.7 ± 1.3	35	- 3.5 ± 1.0	15	0.598

Table S3

Clinical features	TUBA1A N	TUBA1A Percentage	TUBB2B N	TUBB2B Percentage	P-Value
**Epilepsy**	54/82	65.9 %	17/31	54.8 %	0.286
**Microcephaly**	84/113	74.3 %	29/43	67.4 %	0.425
• Primary microcephaly	45/51*	88.2 %*	5/10*	50.0 %*	0.012*
**Developmental delay**	94/95	98.9 %	39/39	100.0 %	1.0
• Global	88/92*	95.7 %*	23/30*	76.7 %*	0.005*
• Cognitive	97/99	98.0 %	31/32	96.9 %	0.572
• Speech	91/92*	98.9 %*	31/35*	88.6 %*	0.020*
• Motor	90/92*	97.8 %*	31/37*	83.8 %*	0.007*
• Isolated learning difficulties	1/91*	1.1 %*	5/35*	14.3 %*	0.006*
• Behavioral disorder	7/95	7.4 %	3/35	8.6 %	1.0
**Motor impairment**	72/74*	97.3 %*	19/26*	73.1 %*	0.001*
• No impairment	2/70*	2.9 %*	5/26*	19.2 %*	0.012*
• Mild	14/70	20.0 %	8/26	30.8 %	n.s.
• Moderate	26/70	37.1 %	4/26	15.4 %	n.s.
• Severe	28/70	40.0 %	9/26	34.6 %	n.s.
**Abnormal muscular tone**	60/67*	89.6 %*	12/25*	48.0 %*	< 0.001*
• Spastic di-/tetraplegia	32/62	51.6 %	6/17	35.3 %	0.281
• Muscular hypotonia	40/65	61.5 %	6/18	33.3 %	0.059
• Dystonia	5/61	8.2 %	2/17	11.8 %	0.643
**Further neurological features**					
• Ataxia	14/61	8.2 %	5/21	23.8 %	1.0
• Gait abnormality	24/62	38.7 %	6/21	28.6 %	0.444
• Dysarthria	6/60	10.0 %	1/20	5.0 %	0.673
• Dysphonia	1/60	1.7 %	0/20	0.0 %	1.0
• Dysphagia	3/60	5.0 %	0/20	0.0 %	0.569
• Dyspraxia	0/60	0.0 %	2/20	10.0 %	0.060
• Facial diplegia	13/60*	21.7 %*	0/20*	0.0 %*	0.031*
• Hearing loss	1/61	1.6 %	1/21	4.8 %	0.449
• Tremor	1/60	1.7 %	2/20	10.0 %	0.153
**Musculoskeletal features**	7/59	11.9 %	6/17	35.3 %	0.061
• Scoliosis	6/58	10.3 %	5/17	29.4 %	0.111
• Arthrogriposis multiplex congenita	0/58	0.0 %	1/17	5.9 %	0.094
**Ocular features**	41/70	58.6 %	15/23	65.2 %	0.631
• Reduced vision	11/66	16.7 %	7/19	36.8 %	0.061
• Microphthalmia	2/66	3.0 %	0/19	0.0 %	1.0
• Exophthalmia	3/67	4.5 %	0/19	0.0 %	1.0
• Other	2/66	3.0 %	0/18	0.0 %	-
• **Ocular motility disorders**					
• Strabismus	22/66	33.3 %	6/18	33.3 %	1.0
• Nystagmus	10/68	14.7 %	3/18	16.7 %	1.0
• Oculomotor apraxia	2/67	3.0 %	0/18	0.0 %	1.0
• CFEOM	0/66*	0.0 %*	4/18*	22.2 %*	0.002*
**Facial dysmorphisms**	40/87	46.0 %	9/24	37.5 %	0.495

Table S4

Region and type of facial dysmorphism	N TUBA1A	N TUBB2B
**Mouth/Jaw**	**35**	**3**
• (Micro-) Retrognatism	15	0
• Downturned corners of the mouth	0	3
• Large mouth	1	0
• Thin lips	3	0
• Everted lower lip	2	0
• Cupid bow shaped upper lip	1	0
• Short/smooth philtrum	3	0
• Wide spaced teeth	3	0
• Microglossia	1	0
• High-arched/cleft palate	5	0
• Unspecified	1	0
**Eyes**	**23**	**1**
• Hypertelorism	9	0
• Hypotelorism	1	0
• Epicanthus	6	0
• Up-/Downslanting palpebral fissures	4	1
• Long eyelashes	2	0
• Thick eyebrows	1	0
**Nose**	**9**	**2**
• Prominent	1	0
• Bulbous nasal tip	2	0
• Anteverted nares	1	1
• Nasal bridge	5	1
○ High	1	1
○ Low	1	0
○ Narrow	1	0
○ Broad, flat	2	0
**Ears**	**9**	**1**
• Macrotia	4	0
• Large earlobes	2	0
• Low set ears	1	0
• Simplified anthelix	1	0
• Dysmorphic helices	0	1
• Unspecified	1	0
**Forehead**	**7**	**1**
• Narrow	1	0
• Broad	1	0
• Sloping	2	1
• Flat	1	0
• High	1	0
• Metopic ridge	1	0
**Further regions**	**2**	**0**
• Short neck	1	0
• Flattening occiput	1	0
**Unspecified dysmorphism**	**8**	**3**

Table S5

	TUBA1A				TUBB2B			
	**Alive**	**Fetuses**	**Deceased**	**Total**	**Alive**	**Fetuses**	**Deceased**	**Total**
**Cortical findings**	84/97<br>86.6 %	24/24<br>100.0 %	5/5<br>100.0 %	113/126<br>89.7 %	35/36<br>97.2 %	7/7<br>100.0 %	2/2<br>100.0 %	44/45<br>97.8 %
**Lissencephaly**	33/96<br>34.4 %*	6/24<br>25.0 %	2/5<br>40.0 %	41/125<br>32.8 %*	1/35<br>2.9 %*	2/7<br>28.6 %	0/2<br>0.0 %	3/44<br>6.8 %*
**Agyria**	23/95<br>24.2 %*	13/24<br>54.2 %	3/5<br>60.0 %	39/124<br>31.5 %*	1/35<br>2.9 %*	0/7<br>0.0 %	0/2<br>0.0 %	1/44<br>2.3 %*
**Pachygyria**	38/95<br>40.0 %*	0/24<br>0.0 %	1/5<br>20.0 %	39/124<br>31.5 %*	2/35<br>5.7 %*	1/7<br>14.3 %	1/2<br>50.0 %	4/44<br>9.1 %*
**Microlissencephaly**	3/95<br>3.2 %	12/24<br>50.0 %	1/5<br>20.0 %	16/124<br>12.9 %	0/35<br>0.0 %	1/7<br>14.3 %	0/2<br>0.0 %	1/44<br>2.3 %
**Simplified gyral pattern**	6/95<br>6.3 %	0/24<br>0.0 %	0/5<br>0.0 %	6/124<br>4.8 %	4/35<br>11.4 %	0/7<br>0.0 %	0/2<br>0.0 %	4/44<br>9.1 %
**Polymicrogyria**	17/94<br>18.1 %*	4/24<br>16.7 %	2/5<br>40.0 %	23/123<br>18.7 %*	16/35<br>45.7 %*	3/7<br>42.9 %	1/2<br>50.0 %	20/44<br>45.5 %*
• Generalized	2/95<br>2.1 %*	2/24<br>8.3 %	0/5<br>0.0 %	4/124<br>3.2 %*	4/35<br>11.4 %*	2/7<br>28.6 %	0/2<br>0.0 %	6/44<br>13.6 %*
• Multifocal	3/95<br>3.2 %*	0/24<br>0.0 %	0/5<br>0.0 %	3/124<br>2.4 %*	9/35<br>25.7 %*	0/7<br>0.0 %	1/2<br>50.0 %	10/44<br>22.7 %*
• Focal	13/96<br>13.5 %	2/24<br>8.3 %	2/5<br>40.0 %	17/125<br>13.6 %	3/35<br>8.6 %	1/7<br>14.3 %	0/2<br>0.0 %	4/44<br>9.1 %
**Polymicrogyria-like cortical dysplasia**	3/95<br>3.2 %*	0/24<br>0.0 %	1/5<br>20.0 %	4/124<br>3.2 %*	9/35<br>25.7 %*	1/7<br>14.3 %	0/2<br>0.0 %	10/44<br>22.7 %*
**Schizencephaly**	0/95<br>0.0 %	0/24<br>0.0 %	0/5<br>0.0 %	0/124<br>0.0 %	1/35<br>2.9 %	0/7<br>0.0 %	0/2<br>0.0 %	1/44<br>2.3 %
**Subcortical band heterotopia**	2/95<br>2.1 %	0/24<br>0.0 %	0/5<br>0.0 %	2/124<br>1.6 %	1/35<br>2.9 %	0/7<br>0.0 %	0/2<br>0.0 %	1/44<br>2.3 %
**Unspecified**	6/96<br>6.3 %	1/23<br>4.2 %	0/5<br>0.0 %	7/125<br>5.6 %	5/35<br>14.3 %	0/7<br>0.0 %	1/2<br>50.0 %	6/44<br>13.6 %

Table S6

	TUBA1A				TUBB2B			
	**Alive**	**Fetuses**	**Deceased**	**Total**	**Alive**	**Fetuses**	**Deceased**	**Total**
**Extra-cortical findings**	105/105<br>100.0 %	24/24<br>100.0 %	5/5<br>100.0 %	134/134<br>100.0 %	36/37<br>97.3 %	7/7<br>100.0 %	2/2<br>100.0 %	45/46<br>97.8 %
**Region and type of abnormality**								
**Cerebellum**	**75/94<br>79.8 %**	**24/24<br>100.0 %**	**5/5<br>100.0 %**	**104/123<br>84.6 %**	**30/36<br>83.3 %**	**7/7<br>100.0 %**	**2/2<br>100.0 %**	**39/45<br>86.7 %**
• Hemispheric dysplasia	19/94<br>20.2 %	10/24<br>41.7 %	1/5<br>20.0 %	30/123<br>24.4 %	5/36<br>13.9 %	1/7<br>14.3 %	1/2<br>50.0 %	7/45<br>15.6 %
• Hemispheric hypoplasia	13/93<br>14.0 %	18/24<br>75.0 %	5/5<br>100.0 %	36/122<br>29.5 %	8/36<br>22.2 %	2/7<br>28.6 %	2/2<br>100.0 %	12/45<br>26.7 %
• Hemispheric agenesis	2/92<br>2.2 %	0/24<br>0.0 %	0/5<br>0.0 %	2/121<br>1.7 %	0/36<br>0.0 %	0/7<br>0.0 %	0/2<br>0.0 %	0/45<br>0.0 %
• Vermian dysplasia	28/94<br>29.8 %	11/24<br>45.8 %	0/4<br>0.0 %	39/122<br>32.0 %	13/36<br>36.1 %	1/7<br>14.3 %	1/2<br>50.0 %	15/45<br>33.3 %
• Vermian hypoplasia	43/94<br>45.7 %	17/24<br>70.8 %	2/4<br>50.0 %	62/122<br>50.8 %	19/36<br>52.8 %	7/7<br>100.0 %	2/2<br>100.0 %	28/45<br>62.2 %
**Corpus callosum**	**94/99<br>94.9 %***	**24/24<br>100.0 %**	**4/4<br>100.0 %**	**122/127<br>96.1 %***	**25/33<br>75.8 %***	**6/7<br>85.7 %**	**n.a.**	**31/40<br>77.5 %***
• Partial agenesis	21/97<br>21.6 %	2/24<br>8.0 %	0/4<br>0.0 %	23/125<br>18.4 %	3/33<br>9.1 %	1/7<br>14.3 %	n.a.	4/40<br>10.0 %
• Complete agenesis	18/98<br>18.4 %	19/24<br>79.2 %	4/4<br>100.0 %	41/126<br>32.5 %	7/33<br>21.2 %	5/7<br>71.4 %	n.a.	12/40<br>30.0 %
• Hypoplasia	51/98<br>52.0 %	2/24<br>8.0 %	0/4<br>0.0 %	53/126<br>42.1 %	16/33<br>48.5 %	1/7<br>14.3 %	n.a.	17/40<br>42.5 %
• Dysplasia	19/98<br>19.4 %	3/24<br>13.0 %	0/4<br>0.0 %	22/126<br>17.5 %	2/33<br>6.1 %	0/7<br>0.0 %	n.a.	2/40<br>5.0 %
**Basal ganglia**	**56/87<br>64.4 %***	**12/20<br>60.0 %**	**2/3<br>66.7 %**	**70/110<br>63.6 %***	**29/34<br>85.3 %***	**4/5<br>80.0 %**	**n.a.**	**33/39<br>84.6 %***
• Dysplasia	56/87<br>64.4 %*	9/20<br>45.0 %	2/3<br>66.7 %	67/110<br>60.9 %*	29/34<br>85.3 %*	3/5<br>60.0 %	n.a.	32/39<br>82.1 %*
• Agenesis	0/87<br>0.0 %	3/20<br>15.0 %	0/3<br>0.0 %	3/110<br>2.7 %	0/34<br>0.0 %	1/5<br>20.0 %	n.a.	1/39<br>2.6 %
• Hypertrophy	4/87<br>4.6 %	0/20<br>0.0 %	1/3<br>33.3 %	5/110<br>4.5 %	0/34<br>0.0 %	0/5<br>0.0 %	n.a.	0/39<br>0.0 %
**Capsula interna**	**60/84<br>71.4 %**	**13/20<br>65.0 %**	**1/3<br>33.3 %**	**74/107<br>69.2 %**	**21/27<br>77.8 %**	**3/4<br>75.0 %**	**n.a.**	**24/31<br>77.4 %**
**Pons**	**30/85<br>35.3 %**	**20/24<br>83.3 %**	**1/4<br>25.0 %**	**51/113<br>45.1 %**	**11/24<br>45.8 %**	**4/5<br>80.0 %**	**1/1<br>100.0 %**	**16/30<br>53.3 %**
• Hypoplasia	26/85<br>30.6 %	18/24<br>75.0 %	1/4<br>25.0 %	45/113<br>39.8 %	10/24<br>41.7 %	4/5<br>80.0 %	1/1<br>100.0 %	15/30<br>50.0 %
• Dysplasia	9/85<br>10.6 %	2/24<br>8.0 %	0/4<br>0.0 %	11/113<br>9.7 %	1/25<br>4.0 %	0/5<br>0.0 %	n.a.	1/30<br>3.3 %
**Brainstem**	**36/87<br>41.4 %**	**15/24<br>62.5 %**	**2/4<br>50.0 %**	**53/115<br>46.1 %**	**14/25<br>56.0 %**	**3/6<br>50.0 %**	**n.a.**	**17/31<br>54.8 %**
• Hypoplasia	28/88<br>31.8 %*	14/24<br>58.3 %	2/3<br>66.7 %	44/115<br>38.3 %	14/25<br>56.0 %*	3/6<br>50.0 %	n.a.	17/31<br>54.8 %
• Dysplasia	9/88<br>10.2 %	2/24<br>8.0 %	1/3<br>33.3 %	12/115<br>10.4 %	0/25<br>0.0 %	0/6<br>0.0 %	n.a.	0/31<br>0.0 %
**Hippocampus**	**25/81<br>30.9 %**	**12/17<br>70.6 %**	**1/4<br>25.0 %**	**38/102<br>37.3 %**	**4/13<br>30.8 %**	**1/2<br>50.0 %**	**n.a.**	**5/15<br>33.3 %**
• Hypoplasia	3/81<br>3.7 %	0/17<br>0.0 %	0/4<br>0.0 %	3/102<br>2.9 %	1/13<br>7.7 %	0/2<br>0.0 %	n.a.	1/15<br>6.7 %
• Dysplasia	22/81<br>27.2 %	12/17<br>70.6 %	1/4<br>25.0 %	35/102<br>34.3 %	4/13<br>30.8 %	1/2<br>50.0 %	n.a.	5/15<br>33.3 %
**Thalamus dysplasia**	**16/81<br>19.8 %**	**5/21<br>22.7 %**	**2/4<br>50.0 %**	**23/107<br>21.5 %**	**4/13<br>30.8 %**	**1/1<br>100.0 %**	**n.a.**	**5/14<br>35.7 %**
**Olfactory bulb agenesis**	**1/80<br>1.3 %**	**9/17<br>52.9 %**	**1/4<br>25.0 %**	**11/101<br>10.9 %**	**0/15<br>0.0 %**	**3/3<br>100.0 %**	**n.a.**	**3/18<br>16.7 %**
**Optical nerve atrophy**	**9/88<br>10.2 %**	**2/20<br>10.0 %**	**1/4<br>25.0 %**	**12/112<br>10.7 %**	**4/17<br>23.5 %**	**0/3<br>0.0 %**	**1/1<br>100.0 %**	**5/21<br>23.8 %**
**Cranial nerve V hypoplasia**	**2/80<br>2.5 %**	**0/21<br>0.0 %**	**0/4<br>0.0 %**	**2/105<br>1.9 %**	**1/13<br>7.7 %**	**n.a.**	**n.a.**	**1/13<br>7.7 %**
**Hypomyelination**	**15/88<br>17.0 %**	**3/22<br>13.6 %**	**2/4<br>50.0 %**	**20/114<br>17.5 %**	**5/16<br>31.3 %**	**0/1<br>0.0 %**	**n.a.**	**5/17<br>29.4 %**
**Ventriculomegaly**	**41/88<br>46.6 %***	**8/23<br>34.8 %**	**2/4<br>50.0 %**	**51/115<br>44.3 %***	**18/21<br>85.7 %***	**4/4<br>100.0 %**	**n.a.**	**22/25<br>88.0 %***

Table S7

	TUBA1A		TUBB2B		
**Histopathological findings**	**N**	**Percentage**	**N**	**Percentage**	**P-Value**
Neuroglial overmigration	2/21	9.5 %*	3/6	50.0 %*	0.024*
Heterotopic neurons	19/22	86.4 %	5/6	83.3 %	0.851
Disorganized cortico-spinal tractum	12/20	60.0 %	1/5	20.0 %	0.109
Enlarged germinal zones	10/21	47.6 %	2/3	66.7 %	0.537
Thin/absent cortical plate	12/20	60.0 %	2/5	40.0 %	0.420
Non-individualized hippocampus	10/19	52.6 %	1/3	33.3 %	0.534
Hypoplastic olivary nuclei	13/20	65.0 %	1/5	20.0 %	0.070
Thick cortex w/ reduced layering	9/20	45.0 %	2/5	40.0 %	0.840
